# Supplementary material for: Thriving under Salinity: Growth, Ecophysiology and Proteomic Insights into the Tolerance Mechanisms of Obligate Halophyte Suaeda fruticosa
Source: Plants (Basel). 2024 May 31;13(11):1529. doi: 10.3390/plants13111529 (PMC11174735; doi:10.3390/plants13111529)
Supplement: Supplementary file 1 [file plants-13-01529-s001.zip › plants-3020385-supplementary.pdf]

Protein	Top Hit			Expression		
	Organism	Acc No	% Coverage	N	O	H
**Amino acid synthesis**						
5-methyltetrahydropteroyltriglutamate--homocysteine methyltransferase	Mesembryanthemum crystallinum	P93263	14.25	39	61	42
Cysteine synthase, chloroplastic/chromoplastic	Spinacia oleracea	P32260	13.58	10	8	5
Serine hydroxymethyltransferase, mitochondrial	Arabidopsis thaliana	Q9SZJ5	9.86	2	3	3
Glutamine synthetase leaf isozyme, chloroplastic	Medicago sativa	Q9XQ94	7.01	3	2	4
Adenosylhomocysteinase	Triticum aestivum	P32112	5.15	7	16	14
Aspartate aminotransferase, cytoplasmic	Daucus carota	P28734	4.44	20	13	8
Ferredoxin-dependent glutamate synthase, chloroplastic	Spinacia oleracea	Q43155	1.91	0	4	1
**Carbohydrate metabolism**						
Glyceraldehyde-3-phosphate dehydrogenase, cytosolic	Antirrhinum majus	P25861	32.94	122	58	88
Malate dehydrogenase, cytoplasmic	Beta vulgaris	Q9SML8	28.01	87	49	71
Enolase 2	Saccharomyces cerevisiae	P00925	24.03	42	14	29
Phosphoglycerate kinase, chloroplastic	Nicotiana tabacum	Q42961	19.13	13	9	10
Phosphoglycerate kinase, cytosolic	Nicotiana tabacum	Q42962	19.02	20	19	18
Fructose-bisphosphate aldolase 1, chloroplastic (Fragment)	Pisum sativum	Q01516	18.26	23	15	24

Protein	Top Hit			Expression		
	Organism	Acc No	% Coverage	N	O	H
Triosephosphate isomerase, cytosolic	Oryza sativa subsp. japonica	P48494	17.39	22	10	3
Phosphoglucomutase, cytoplasmic	Populus tremula	Q9ZSQ4	10.31	8	3	5
Transketolase, chloroplastic	Spinacia oleracea	O20250	9.72	13	9	8
UTP--glucose-1-phosphate uridylyltransferase	Pyrus pyrifolia	O64459	8.49	2	3	8
Sedoheptulose-1,7-bisphosphatase, chloroplastic	Arabidopsis thaliana	P46283	6.87	3	2	3
UDP-arabinopyranose mutase 1	Arabidopsis thaliana	Q9SRT9	6.16	2	1	4
Glucose-1-phosphate adenylyltransferase small subunit, chloroplastic/amyloplastic	Hordeum vulgare	P55238	5.85	5	3	1
Phosphoenolpyruvate carboxylase 1	Zea mays	P04711	4.02	44	30	12
Granule-bound starch synthase 1, chloroplastic/amyloplastic	Pisum sativum	Q43092	3.98	10	2	3
Pyruvate kinase 1	Saccharomyces cerevisiae	P00549	20.2	15	5	15
Pyruvate decarboxylase isozyme 1	Saccharomyces cerevisiae	P06169	12.43	20	9	18
Isocitrate dehydrogenase [NADP], chloroplastic (Fragment)	Medicago sativa	Q40345	6.47	3	2	3
**Cofactor/ Coenzyme synthesis**						
S-adenosylmethionine synthase 1	Atriplex nummularia	Q7XZR1	35.35	69	81	117

Protein	Top Hit			Expression		
	Organism	Acc No	% Coverage	N	O	H
Thiamine-phosphate pyrophosphorylase	Streptococcus suis	A4W0K4	15.28	2	3	4
Thiazole biosynthetic enzyme, chloroplastic	Arabidopsis thaliana	Q38814	9.74	2	2	1
4-hydroxythreonine-4-phosphate dehydrogenase	Geobacter lovleyi	B3E252	11.9	5	4	4
**Defense**						
Putative germin-like protein subfamily 1 member 9	Arabidopsis thaliana	Q9FMB0	13.96	5	2	1
**Detoxifying and antioxidant**						
Glutathione S-transferase class-mu 26 kDa isozyme	Schistosoma japonicum	P08515	29.36	112	20	4
Thioredoxin H-type 1	Nicotiana tabacum	P29449	17.46	2	3	1
Peroxisomal (S)-2-hydroxy-acid oxidase GLO1	Arabidopsis thaliana	Q9LRR9	6.27	8	10	8
Catalase-3	Arabidopsis thaliana	Q42547	5.08	5	1	5
**DNA modifying**						
Probable histone H2A.1	Medicago truncatula	Q2HU68	35.14	57	8	4
Histone H4	Xenopus borealis	P62798	33.01	15	1	0
Histone H2B.5	Triticum aestivum	Q43216	19.12	17	6	0
**Energy production and conversion**						
ATP synthase subunit beta, chloroplastic	Magnolia tripetala	Q9MU41	54.82	149	154	133
ATP synthase epsilon chain, chloroplastic	Eucalyptus globulus subsp. globulus	Q49KZ2	24.06	5	6	7

Protein	Top Hit			Expression		
	Organism	Acc No	% Coverage	N	O	H
ATP synthase subunit alpha, chloroplastic	Silene latifolia	Q589B3	19.72	42	52	44
V-type proton ATPase subunit B2	Arabidopsis thaliana	Q9SZN1	8.21	27	20	31
Alcohol dehydrogenase class-3	Zea mays	P93629	7.87	2	0	3
ADP,ATP carrier protein 1, mitochondrial	Triticum aestivum	Q41629	7.55	5	2	4
ATP synthase gamma chain 1, chloroplastic	Arabidopsis thaliana	Q01908	6.7	2	2	3
Formate--tetrahydrofolate ligase	Spinacia oleracea	P28723	5.81	3	2	3
**Photosynthesis**						
Alanine aminotransferase 2	Hordeum vulgare	P52894	14.11	18	18	19
Glutamate-1-semialdehyde 2,1-aminomutase	Synechococcus sp.	Q3AWP4	6.54	2	4	7
NAD(P)H-quinone oxidoreductase subunit I, chloroplastic	Spinacia oleracea	Q9M3I7	21.18	5	2	4
Pyruvate, phosphate dikinase, chloroplastic	Flaveria trinervia	P22221	14.48	99	67	42
Ferredoxin--NADP reductase, leaf isozyme 1, chloroplastic	Arabidopsis thaliana	Q9FKW6	13.06	13	8	11
Photosystem I iron-sulfur center	Illicium oligandrum	A6MMZ6	51.85	32	21	18
Ribulose bisphosphate carboxylase large chain	Daphniphyllum sp.	P28397	51.61	364	380	373

Protein	Top Hit			Expression		
	Organism	Acc No	% Coverage	N	O	H
Chlorophyll a-b binding protein 21, chloroplastic	Nicotiana tabacum	P27493	36.6	23	36	27
Photosystem I reaction center subunit II, chloroplastic	Spinacia oleracea	P12353	35.85	35	24	26
Photosystem I reaction center subunit IV, chloroplastic	Spinacia oleracea	P12354	26.4	5	9	5
Carbonic anhydrase, chloroplastic	Arabidopsis thaliana	P27140	21.61	42	30	39
Apocytochrome f	Nicotiana tabacum	P06449	20.62	22	17	20
Oxygen-evolving enhancer protein 2, chloroplastic	Solanum tuberosum	P93566	18.85	8	16	11
Oxygen-evolving enhancer protein 1, chloroplastic	Spinacia oleracea	P12359	16.57	70	49	50
Cytochrome b6-f complex iron-sulfur subunit, chloroplastic	Spinacia oleracea	P08980	13.04	2	2	3
Oxygen-evolving enhancer protein 3-1, chloroplastic	Zea mays	Q41048	9.22	8	7	12
Geranylgeranyl diphosphate reductase, chloroplastic	Arabidopsis thaliana	Q9CA67	6.85	5	2	3
Photosystem II CP43 chlorophyll apoprotein	Nicotiana sylvestris	Q3C1I3	6.51	12	5	5
RuBisCO large subunit-binding protein subunit beta, chloroplastic	Brassica napus	P21241	4.25	2	6	4
**Post translational modification and signal transduction**						
Peptidyl-prolyl cis-trans isomerase CYP20-3, chloroplastic	Arabidopsis thaliana	P34791	10.77	5	2	3

Protein	Top Hit			Expression		
	Organism	Acc No	% Coverage	N	O	H
ATP-dependent Clp protease ATP-binding subunit clpA homolog	Populus euphratica	P84565	37.5	3	1	3
Heat shock cognate 70 kDa protein 1	Solanum lycopersicum	P24629	20.92	54	35	24
Luminal-binding protein 5	Nicotiana tabacum	Q03685	10.63	8	6	3
Alcohol dehydrogenase 1	Saccharomyces cerevisiae	P00330	7.18	7	1	3
Cell division cycle protein 48 homolog	Capsicum annuum	Q96372	7.08	5	8	10
Chaperone protein ClpC1, chloroplastic	Arabidopsis thaliana	Q9FI56	4.31	5	1	1
Chaperone protein DnaK	Sphingomonas wittichii	A5V5P9	3.81	3	4	4
GTP-binding protein ypt71	Schizosaccharomyces pombe	Q9HDY0	25	15	2	0
**Transcription and translation**						
Elongation factor Tu, chloroplastic	Arabidopsis thaliana	P17745	7.35	2	2	0
40S ribosomal protein S12	Saccharomyces cerevisiae	P48589	31.47	5	1	1
60S ribosomal protein L12-3	Arabidopsis thaliana	Q9FF52	26.51	8	7	11
Elongation factor 1-alpha	Saccharomyces cerevisiae	P02994	19.65	174	62	144
31 kDa ribonucleoprotein, chloroplastic	Nicotiana sylvestris	P19683	15.56	5	6	4

Protein	Top Hit			Expression		
	Organism	Acc No	% Coverage	N	O	H
Aspartyl-tRNA synthetase 1	Syntrophus aciditrophicus	Q2LPW5	4.43	2	2	1
Elongation factor 2	Saccharomyces cerevisiae	P32324	4.28	13	0	4
**Transport and structural**						
Tubulin alpha-2/alpha-4 chain	Arabidopsis thaliana	P29510	19.78	10	13	18
Actin-46 (Fragment)	Solanum tuberosum	P93586	7.74	8	8	7
Tubulin beta-1 chain (Fragment)	Avena sativa	P25862	34.97	18	16	15
